# Supplementary figures and images for: Construction of a fiber-optically connected MEG hyperscanning system for recording brain activity during real-time communication
Source: PLoS One. 2022 Jun 23;17(6):e0270090. doi: 10.1371/journal.pone.0270090 (PMC9223398; doi:10.1371/journal.pone.0270090)

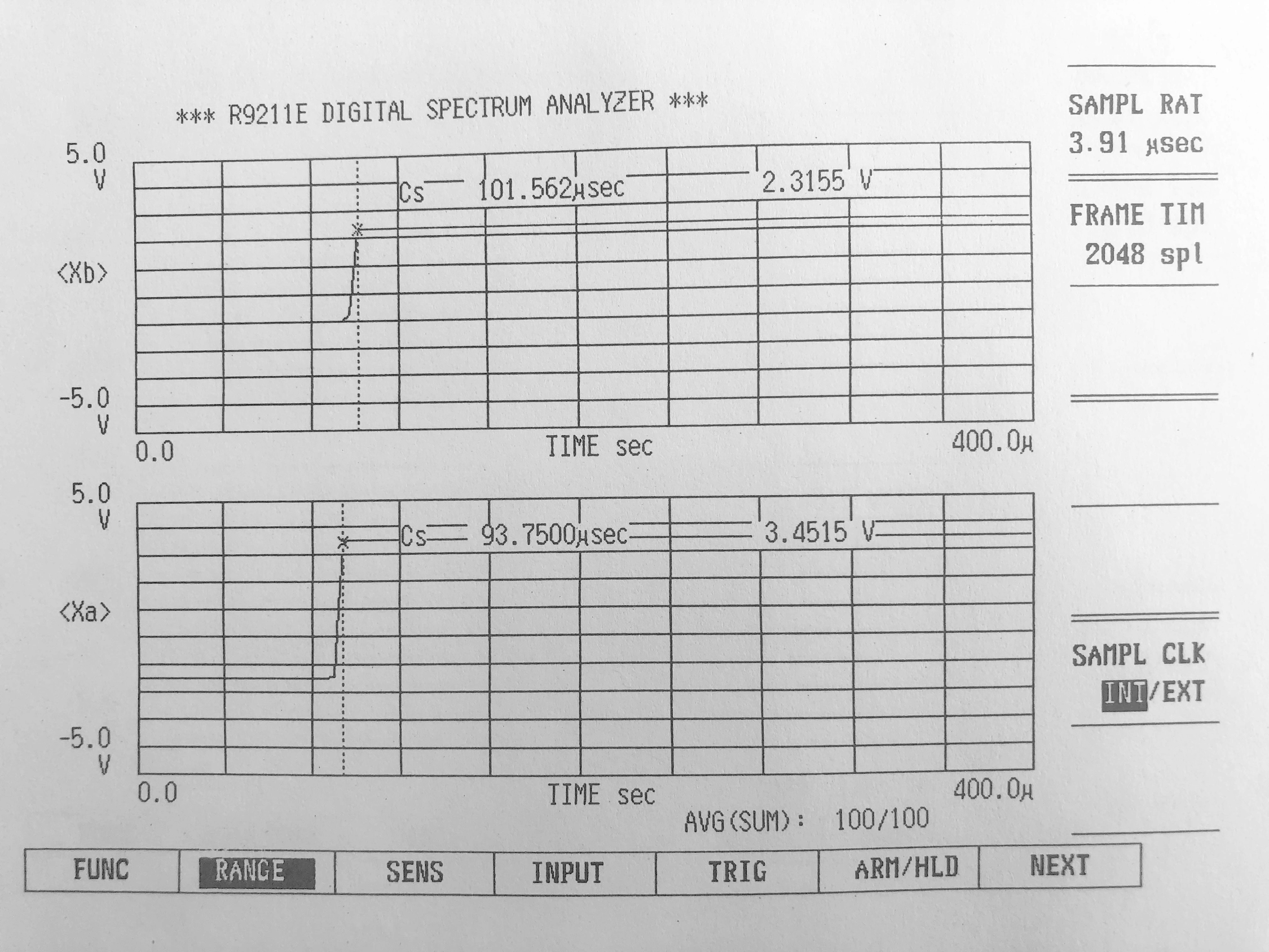

Supplement: S1 Fig — TTL measured by a digital spectrum analyzer. Top: loop-back value, bottom: direct measurement value. doi:10.6084/m9.figshare.19127282. (TIF) [file pone.0270090.s001.tif]

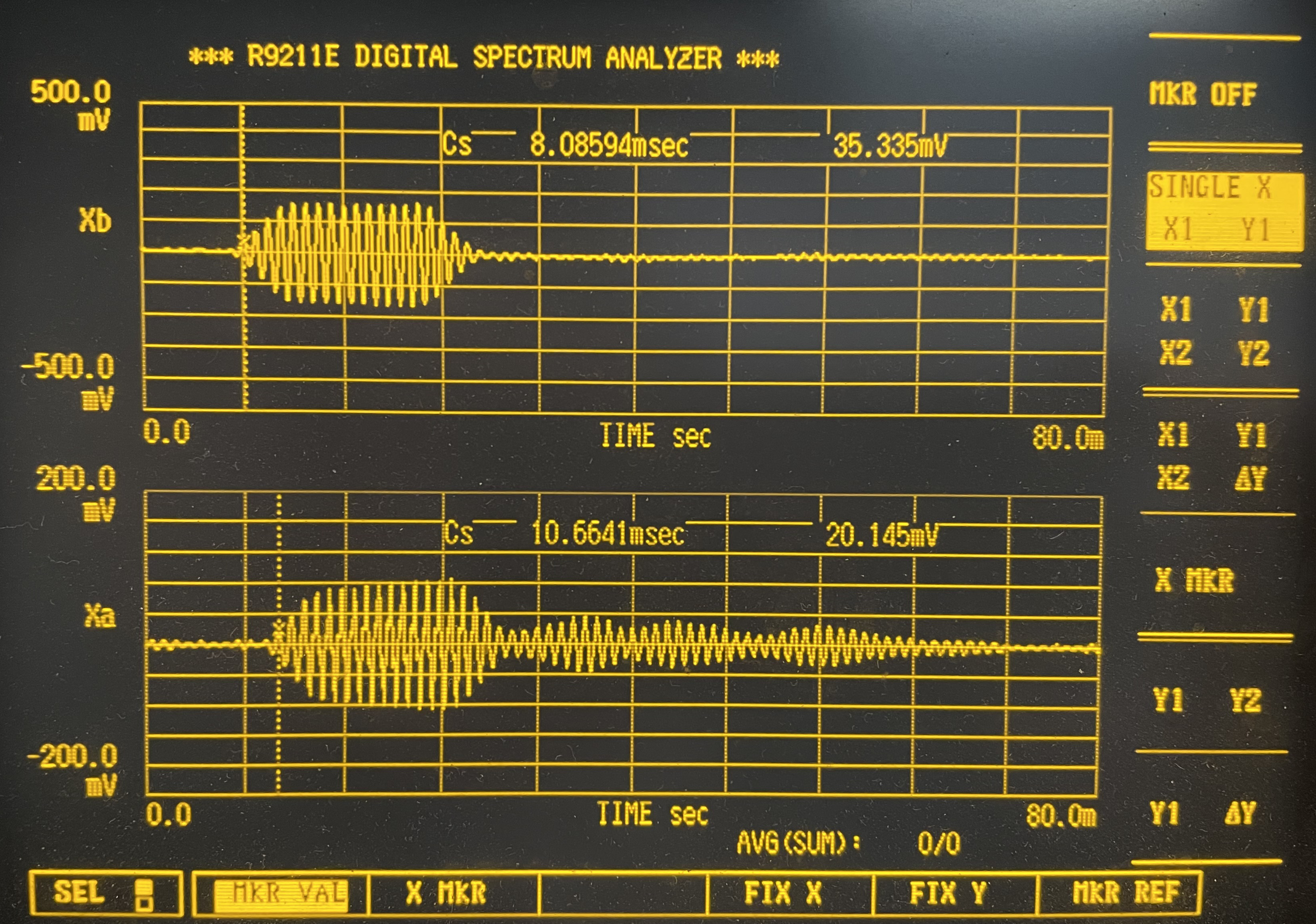

Supplement: S2 Fig — Auditory latency measured by a digital spectrum analyzer. Top: with delay value, bottom: direct measurement value. doi:10.6084/m9.figshare.14872785. (ZIP) [file pone.0270090.s002.zip › hos2res.tif]

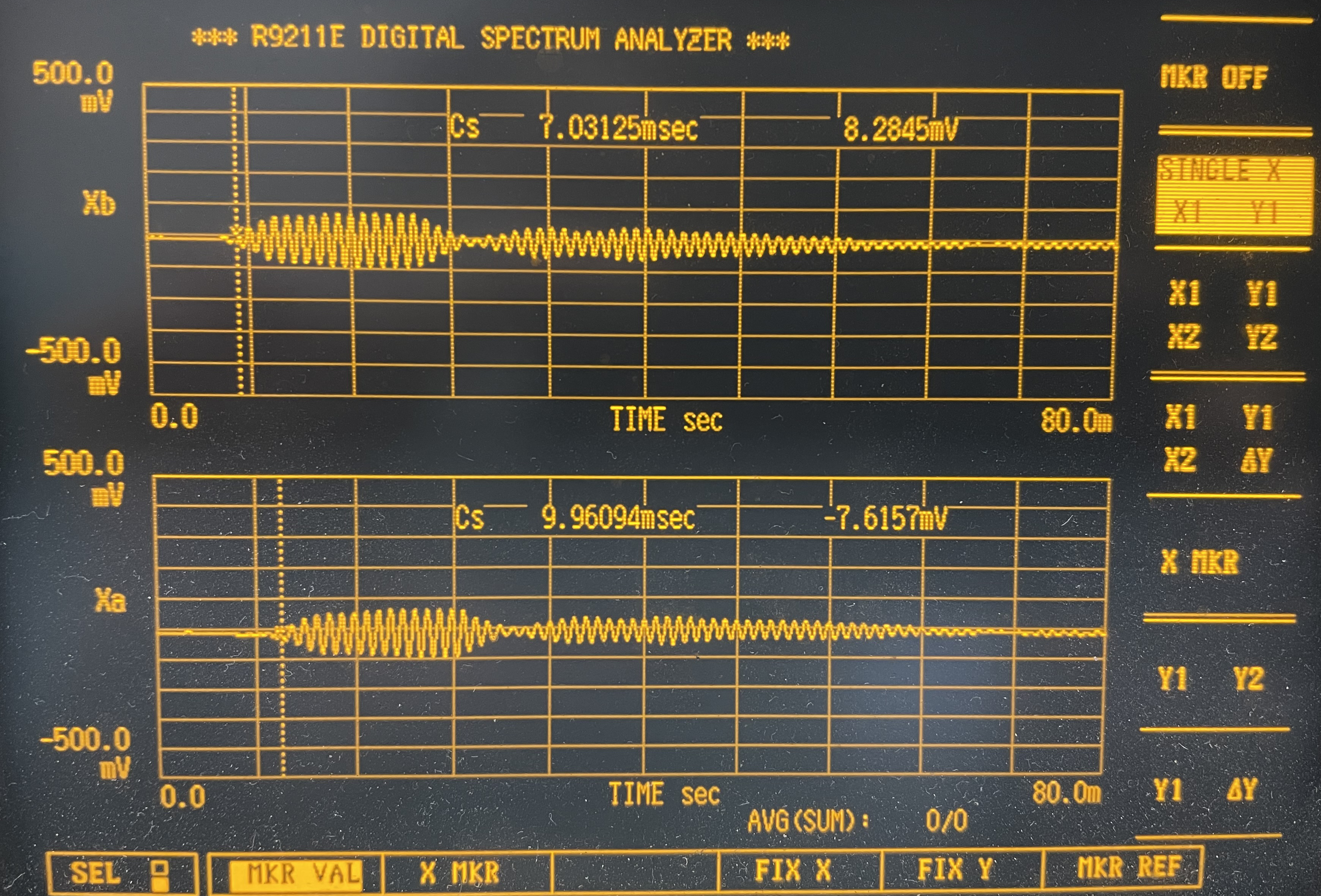

Supplement: S2 Fig — Auditory latency measured by a digital spectrum analyzer. Top: with delay value, bottom: direct measurement value. doi:10.6084/m9.figshare.14872785. (ZIP) [file pone.0270090.s002.zip › res2hos.tif]
