# Supplementary material for: Construction of a fiber-optically connected MEG hyperscanning system for recording brain activity during real-time communication
Source: PLoS One. 2022 Jun 23;17(6):e0270090. doi: 10.1371/journal.pone.0270090 (PMC9223398; doi:10.1371/journal.pone.0270090)
Supplement: S1 Appendix — A proposal for measuring visual evoked field with high accuracy based on jitter and latency of visual signals transmission. (PDF) [file pone.0270090.s005.pdf]

S1 Appendix: Methods for measuring brain activity time-locked to visual signals

The main factors that determine the latency and jitter of the video signal are related to the unsynchronized shutter timing of the camera/projector and the frame rate of the mixer. If higher temporal accuracy is needed, for example to study early stage visual perception, the following solutions can be considered:

1. Adding a marker to each video signal. A prominent optical signal (e.g., flashlight) can be presented at the time of each important event. The marker should be located in an area that is invisible to both participants. If a photo diode picks up the marker at the receiving site, the precise timings of important events can be confirmed.

2. Increasing the frame rate of all devices between the camera and projector. Most of the current delay and jitter were due to the use of a 60-Hz device, with each frame lasting 16.67 ms. By using a high-speed camera and other devices that have the same frame rate as the camera, delay and jitter can be reduced.

3. Adjusting the shutter timing of all devices between the camera and projector. We adjusted for the frame rates of the camera, mixer, and projector in our system. However, the shutter timing was not controlled for any of the devices, and this incongruity causes jitter. Thus, adjusting the shutter timing of all devices should nullify jitter, although minimal latency would remain.
