# Supplementary material for: Construction of a fiber-optically connected MEG hyperscanning system for recording brain activity during real-time communication
Source: PLoS One. 2022 Jun 23;17(6):e0270090. doi: 10.1371/journal.pone.0270090 (PMC9223398; doi:10.1371/journal.pone.0270090)
Supplement: Attachment — Submitted filename: ResponseToReviewers.pdf [file pone.0270090.s007.pdf]

<Response to reviewers>

Reviewer #1: The authors adequately responded to my previous comments. I'm sure that now the authors could get better understanding about the importance of this research project, and about what the authors did to build the hyperscanning MEG system. Now I only have some minor comments.

1.

The authors cited several papers using hyperscanning fMRI system. I recommend that the authors have to select more appropriate papers. First, while the study by Schippers and his colleagues is great, it is not the study of hyperscanning focusing on the interactivity during communication. In this study, two participants could not mutually exchange information. In the case, the video/audio delay does not become a big issue. I suggest that the authors should cite more appropriate literatures investigating neural basis of real social interaction. If the authors think that number of hyperscanning fMRI papers cited in this paper is too much, I strongly suggest some papers by Japanese hyperscanning teams should be replaced by these following papers.

Bilek group

Bilek, E., Ruf, M., Schäfer, A., Akdeniz, C., Calhoun, V. D., Schmahl, C., et al. (2015). Information flow between interacting human brains: identification, validation, and relationship to social expertise. Proc. Natl. Acad. Sci. U.S.A. 112, 5207–5212. doi: 10.1073/pnas.1421831112

Chinese team

https://www.pnas.org/doi/10.1073/pnas.1917407117

Netherland team

https://www.ncbi.nlm.nih.gov/pmc/articles/PMC4280639/

Thank you very much for your useful comment and recommendations. We have carefully reviewed the cited papers and replaced some of them. We have excluded some references which used the same system shown in other reference. The added references are shown below.

Krill AL, Platek SM. Working Together May Be Better: Activation of Reward Centers during a Cooperative Maze Task. PLOS ONE. 2012;7(2):1–7. doi:10.1371/journal.pone.00306.

Stolk A, Noordzij ML, Verhagen L, Volman I, Schoffelen JM, Oostenveld R, et al. Cerebral coherence between communicators marks the emergence of meaning. Proceedings of the National Academy of Sciences. 2014;111(51):18183–18188. doi:10.1073/pnas.141488611.

Bilek E, Ruf M, Sch¨afer A, Akdeniz C, Calhoun VD, Schmahl C, et al. Information flow between interacting human brains: Identification, validation, and relationship to social expertise. Proceedings of the National Academy of Sciences. 2015;112(16):5207–5212. doi:10.1073/pnas.1421831.

Xie H, Karipidis II, Howell A, Schreier M, Sheau KE, Manchanda MK, et al.Finding the neural correlates of collaboration using a three-person fMRI hyperscanning paradigm. Proceedings of the National Academy of Sciences.2020;117(37):23066–23072. doi:10.1073/pnas.191740.

2.

Overall, there is not enough information about what figures show. For example, in Figure 3, the authors showed activation/deactivation (ERS/D) rendered on the surface image. Does the row of surface images in lower panel represent the passage of time? Let us show how the authors calculated the activation on one surface image in detail: which time bin corresponds to the image, how the activation is depicted, whether is this the left hemisphere, and so on. Please also add left-right and AP (anterior-posterior) information. I understand that this small experiment is not the main report of the authors. However, please clearly describe the process that led to these figures and what these figures mean.

Thank you for pointing this out. We have described the process to obtain images in detail in the revised figure caption. Additionally, time bins and orientation of the brain have been added in the figure.

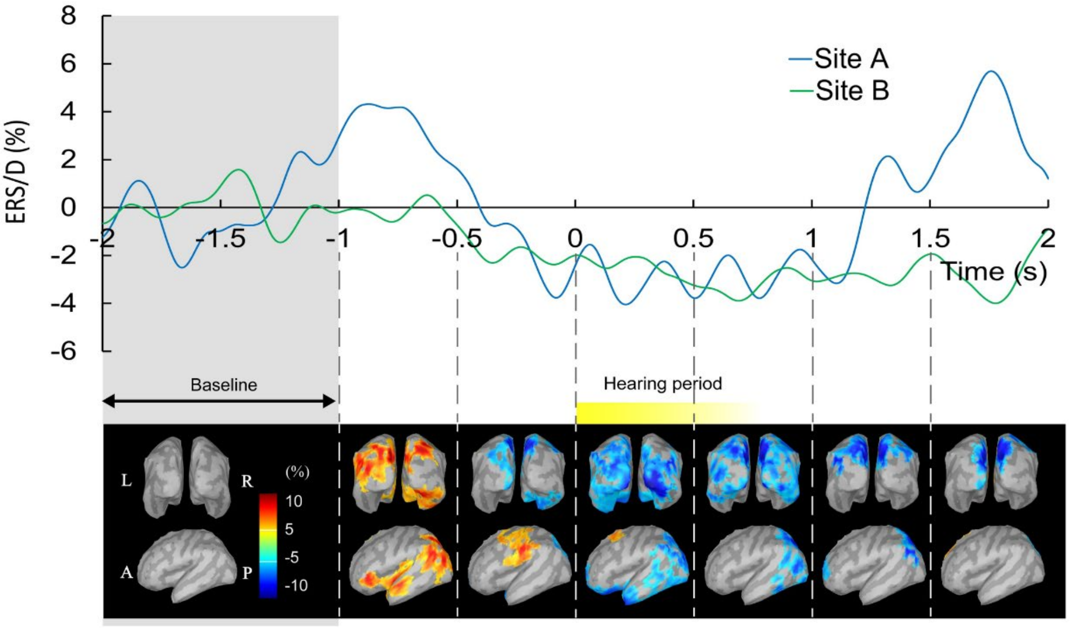

Fig 3. Amplitude modulation of alpha-band rhythm during face-to-face conversation. The brain responses when two subjects faced each other via the A/V devices and spoke words in turns are shown. Mean alpha rhythm amplitude across 128 speech exchanges was normalized by the mean amplitude over the baseline period, from -2 and -1 s, to calculate Event Related Synchronization (ERS) and Event Related Desynchronization (ERD). The time traces of ERS/D averaged over the whole brain of each subject at site A (blue line) and B (green line) are shown on the upper panel. The brain activity of the subjects at both sites reflects that which is associated with listening, with time point 0 ms being the moment of speech onset of the opposite party. ERD of alpha rhythm is exhibited just before and during hearing the speech (mean: 0.7 s) of the opposite party. The brain surface images on the lower part show mean distributions of ERS (red; <+5 %) and ERD (blue; >-5%) on each of the 15,002 vertices across both subjects; back-view (upper row) and left side-view (lower row). This mean alpha rhythm ERS/D was furthermore averaged temporally within each 0.5 s bin. A distinct ERD in the bilateral occipital region (visual area) and left temporal region (linguistic area) observed after 0 s indicate functional involvement of both the visual and auditory systems, suggesting that each subject could visually predict the onset of the opposite party's speech. Abbreviations. L: Left, R: Right, A: Anterior, P: Posterior.

3.

Figures in Supporting information could be described a bit more carefully. See, Figure S1. The authors claimed that 'The loopbacked (round-trip) signal delayed for 7.812 μs (Upper) compared to direct signal (Lower)', however, I have no idea where these information could be found in the TIFF file.

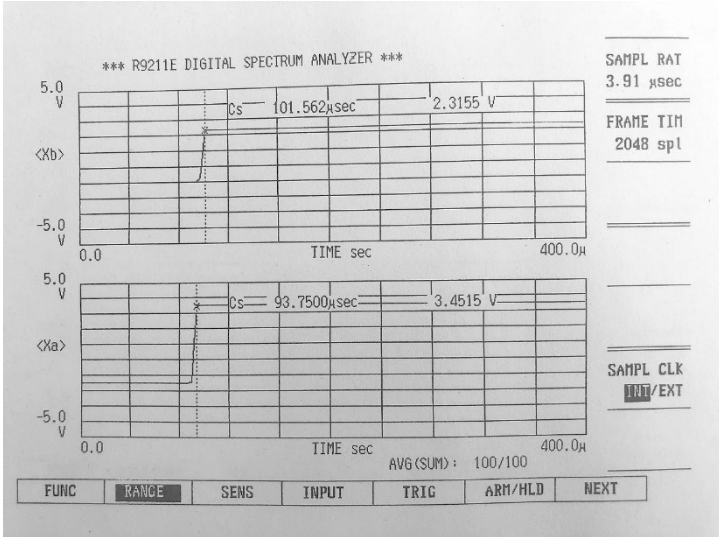

Thank you for the comments. The explanation was too short. We have revised the explanation to describe detail.

Photograph of the digital oscilloscope monitor output showing the TTL signal delay between the two MEG sites (site A: Hokkaido University Medical and Dental Research Building, site B: Hokkaido University Hospital). TTL signal latency was measured by the loopback (round-trip) method, where the signal is sent from site A to site B and back to site A. The dotted line, X mark, and time in each diagram indicate the rising edge of the TTL signal; 101.562 μs in the loopback measurement (upper) and 93.750 μs in the direct measurement (lower). Loopback delay of 7.812 μs was yielded by subtracting these times. One-way TTL signal delay from the site A to the site B was obtained as 3.906 μs, half of the loopback delay. Note that the sampling rate of the digital oscilloscope, 3.91 μs (see the SAMPL RAT in the upper right corner), limited the time resolution.
