# Supplementary material for: Construction of a fiber-optically connected MEG hyperscanning system for recording brain activity during real-time communication
Source: PLoS One. 2022 Jun 23;17(6):e0270090. doi: 10.1371/journal.pone.0270090 (PMC9223398; doi:10.1371/journal.pone.0270090)
Supplement: Attachment — Submitted filename: Response to Reviewers.docx [file pone.0270090.s006.docx]

**<****Reviewers' comments and our responses>**

**Reviewer1**

**In the present paper, the authors reported the details of the Hyperscanning MEG system. Information on how to build up the system, especially information about what devices are used in the integrated system, is usually unclear in the most of hyperscanning systems. Therefore, the present paper would be informative for potential readers who would like to prepare the hyperscanning MEG. However, unfortunately, I have several concerns with this paper.**

**First and the most important thing is that the scientific significance of this paper is unclear. A low latency to transmit signals is very important. I agree. But when the two devices are apart from each other, and connected via some transmitters, the lag cannot be zero. The hyperscanning EEG and NIRS are better in this respect, because it is possible to record two brains using one device, and two participants could do communication directly without any audio/video devices.**

**Potential readers want to know how much better the author’s hyperscanning MEG compared to old-type hyperscanning MEG especially in ability to depict the inter-brain synchrony. The comparison is indispensable if the author would like to stress the advantage of this setting. What kind of inter-brain effect could be specifically observed only by this hyperscanning MEG? How the subtle inter-brain effect (i.e., inter-brain sync degree) could be specifically captured by the system? In this paper, there is no such results, so this is merely a technical paper: ‘I found new better device, so I integrated it on the recording system. It is better than previous one, because the device is new. I have no idea how the new device contribute to the recording system’. That is the only message I could receive from the paper.**

**For example, the authors could make clear how the small delay, that was in the old-fashioned hyperscanning MEG but not in this present system, affects the detection of inter-brain sync, by doing a small experiment.**

**Because not all hyperscanning MEG system could not be connected via fiber optics, so they are almost always ignoring the delay effect. It would be useful if the authors declare what problems can occurs when there is small latency. Of course, there is nothing wrong with reporting this as a technical report. But I don't think it is a scientific paper.**

Thank you for these important comments. As you mentioned, there is no latency in intermediate device-free systems consisting of EEGs or NIRSs. However, they suffer from low spatial resolution. NIRS in particularly is completely incapable of detecting deep brain activities and has low temporal resolution. We believe that these systems are inferior to the combined temporal and spatial resolution of MEG to explore undiscovered communication-related brain functions.

Furthermore, even in EEG or NIRS based systems, intermediate devices are sometimes required to realize experimental protocols or for precise control of conditions condition. For example, Takano et al. (2020) compared brain activities between when subjects viewed a face in real-time and when they viewed a pre-recorded video of a face. Intermediate video/audio devices were essential for these experiments. In simple face-to-face set-ups, there might be no latency, but the trade offs are major constrains in experimental designs.

MEGs have high spatial and temporal resolutions. Our system consisting of two MEGs connected with minimum latency permits the realization of new neurophysiological and psychological protocols.

Although the latencies in our system may be insufficient to detect inter-brain synchronization in experiments based on audiovisual stimuli, that in no way precludes our system’s ability to observe essential brain activities of communication. Indeed, we did not design our system with the intention to analyze inter-brain synchronization during face-to-face communication, but to realize natural face-to-face communication without any discomforts in an MEG-based hyperscanning system. Natural communication was the primary requisite. Of the previously reported MEG hyperscanning systems the highest performer from a visual transmission perspective had a visual signal delay in the range of 100-160 ms excluding delay by the Internet (Zhdanov et al., 2016). Our system achieved a visual signal delay of less than 100 ms and can synchronize audio signals to match the delay of the visual signals– thereby permitting natural communication.

That said, it is possible to further optimize our system to detect inter-brain synchronization, the details regarding which are described in the appendix. For now, we have added simple physiological experimental results obtained by this system in response to comments by Reviewer 2. Methods, Results, and Discussion sections have been modified accordingly (see also response to Reviewer 2).

The following sentence has been added in the Conclusions section L358 to clarify our argument.

L358:

Finally, we would like to highlight the importance of the intermediate devices used to transmit/receive audiovisual signals in hyperscanning systems. The quality of these devices and the validation of their signal processing latencies and characteristics is essential for realizing well-controlled experimental designs in neuropsychophysiological experimentation. Moreover, the minimization of the latency through these intermediate devices, such as via a direct fiber optic connection, is a fundamental priority for hyperscanning research protocols in any modality, not only MEG.

**It is difficult to understand why latency needs to be calculated for reference signals. I understood that the authors want to separate the latency caused by the transmission line (mainly fiber optics) from the latency caused by the microphones and speakers. However, I don't know if my understanding is correct since nothing is mentioned in the paper. Please explain clearly why you need to do this measurement.**

The reference signals were used to determine the base latency of transmission, which is needed to accurately measure and interpret the overall latency of our system, which included the transmission line. Essentially, we just verified that the latency of the reference signals were short enough to be considered zero.

We changed the sentence from L143 to clarify this point.

L143:

The latency of video or audio signals caused by conversion, transmission, and passage of the signal through all intermediate devices. To measure the latencies of the video or audio signal from one site to the other, references were required to know the onset time of the signal. The reference signals also had inherent latencies. Therefore, the latencies of the reference signals were also evaluated.

Latencies were measured 100 times with a digital oscilloscope and averaged. Jitter was observable as distortion in the averaged latency. When evaluating jitter, latencies derived via the MEG Acqs at a sampling rate of 1,000 Hz were analyzed to determine their mode, average, variance, and range.

**The structure of the paper is really far away from standard style: there is no Results section. I think the Results section might be after L204, but please adjust the format. The unconstructed paper is very hard to read.**

Thank you. We added “Results” (section name) to L204.

**This is an opinion that has nothing to do with the scientific point of view. Please check the contents of the paper carefully by the authors, before submission. Quality of paper is very low. Here are some examples: sentences starting with L1 have a period at the end for some reason;**

We are very sorry, but despite thorough and careful checking, we could not find any ridiculous point in L1. In our copy of the original submitted manuscript, the L1 sentence is just “Introduction” (section heading). The ridiculous text you observed may have been caused by the inadvertent introduction of errors during the editorial process.

**the content of the paragraph starting with L77 is exactly the same as that starting with L57.**

Thank you. We removed one of them.

**I am not a native speaker of English, but I found many points with wrong English grammar. I recommend that the authors send it to an English proofreader before submission.**

Thank you for your suggestion. We have had the paper re-edited by an MEG neuroscientist and native English speaker.

**Minor comments**

**L228: Latency purple?**

It was an unnecessary description. It has been removed (L244).

**L241: What is the ‘mus’?**

It has been corrected to μs (L254).

**L243: How did the authors confirm that there is no distortion of sine-wave?**

It was confirmed by visual inspection. We have now clarified this in the text. Photographs of the waveforms are shown in the S2 File (DOI:10.6084/m9.figshare.14872785).

L255

There was no distortion of the sine wave, based on visual inspection, thus indicating an absence of jitter.

**L70 etc.: The term TTL was repeatedly explained.**

We removed one of them.

**L85: ‘Photos: with permission by the models.’ It is redundant.**

It was an unnecessary description. It has been removed.

**L214: ‘MEG Aqs’ What is the Aqs?**

This refers to MEG data acquisition systems (MEG Acqs), as explained in L62.

**L236: What is the lip-synchronized delay?**

Thank you for pointing out. This term was inappropriate. Based on the comments of Reviewer 2, the Fig 2 legend has been substantially revised. It has therefore been corrected as follows.

L235

**Fig 2. Video and audio signal latency distribution.** Video and audio signal latencies from site A to site B and those from site B to site A are superimposed. Audio signal latencies (red/blue bars on the left) are short and have no jitter, while video signal latencies (red/blue bars on the right) are longer and have some jitter (mean: 76.85 ms, SD: 6.57 ms) ranging from 60--100 ms. To optimize the setup for natural communication, audio signals can be delayed to match the latency of the video signals (white bar).

**L288: The authors said ‘See the Appendix’. Where is your appendix? I could not find it.**

The data can now be found in the S1 Appendix in the revised submission.

**Reviewer2**

**Watanabe et al. developed a hyperscanning system in two MEG devices with a low latency for audio and video communications. The study presented a newly developed hyperscanning system that can provide the potential to study brain dynamics of social interactions. The systematic configuration was well established and the audio-visual latencies were verified for real-time face-to-face communications. I have minor comments to improve the quality of the manuscript.**

**- Audio and visual latencies should be measured from the site B to the site A as well for the bidirectional communication. Further, the effect of the direction of the transmission should be investigated.**

Thank you for this important suggestion. Our MEG hyperscanning system has the same transmitter, receiver, transmission lines, and intermediate devices at both sites. Therefore, we believed there were no directional differences in latency. However, in light of this comment, we made an attempt to confirm that notion, and have appended the results in Fig 1 and revised the text as follows.

L161:

The LED light was flashed 200 times over five sessions (total 1,000 times) at site A. The light was captured by the video camera at site A, and transmitted via all intermediate devices to site B, where the light was projected into the shielded room and detected by a photo diode. A square signal was generated by the output of the photodiode and input into the MEG Acqs at site B. This measurement process was performed in the opposite direction as well.

L177:

The sine wave signal generated by the PC at site A was split. One part was transmitted directly to site B and recorded on a digital oscilloscope. The other part was played on a non-magnetic speaker and sampled by a monaural microphone in the shielded room at site A. The signal captured by this microphone was then transmitted to site B where it, underwent digital audio conversion and passed through the A/V mixer. It was then re-played on a non-magnetic speaker and sampled by a monaural microphone in the shielded room at site B. The sampled signal was recorded on the same digital oscilloscope at site B. The audio waves of the two split signals were compared. This measurement process was performed in the opposite direction as well.

L225:

The latencies of the 1,000 LED light flash at both sites are summarized on a histogram with 2-ms bins (Fig 2, site A blue bars, site B red bars, S1 File). From site A to site B, the mode was 70--72 ms (mean = 76.76 ms, SD = 5.34 ms, and range = 66.42--97.42 ms); from site B to site A, the mode was 76--78 ms (mean = 76.94 ms, SD = 7.61 ms, and range = 63.42--95.42 ms). Here, the transmission takes 2.36 μs, which was calculated by the transmission speed of the HDMI cable 0.5 μs/100 m and cable length of 472 m, and conversion takes 400 μs in total (200 μs for optic I/O module B and C in Fig 1). As both sites had the same devices and set-ups, the latency distributions were nearly identical, ranging from 60 ms to 100 ms. These latencies are sufficiently short for natural communication, thereby meeting the objective of this system.

L242:

The processing latency of one of the intermediate devices, the A/V mixer, is 16.67 ms/frame. This latency is small compared to the mean overall latency of about 77 ms. Hence, the majority of the video latency is implicitly caused by the camera and the projector. The signal transmission of the camera and the projector are 1080p/60p, i.e., one frame equates to 16.67 ms. Jitter was presumed to be caused by the frame of both the camera and the projector, and was therefore calculated as 33.34 ms (16.67 ms × 2 devices). The latency range of our measurement results (about 31.5 ms) closely coincides with this value.

L258:

A comparison of the audio waves of the two split signals demonstrated a constant latency of 3.13 ms (from Site A to Site B) and 2.78 ms (from Site B to Site A) with no jitter (Fig 2, red and blue bar, S2 Fig). The reason for the slight directional difference is not clear, but we suspect that it might depend on the distance between the microphone and the speaker at each site. Regardless, the minute directional difference (0.4 ms) is arguably not physiologically discernible, and the approximately 3 ms jitter-free latency in both directions is sufficiently low for natural communication.

L235:

**Fig 2. Video and audio signal latency distribution.** Video and audio signal latencies from site A to site B and those from site B to site A are superimposed. Audio signal latencies (red/blue bars on the left) are short and have no jitter, while video signal latencies (red/blue bars on the right) are longer and have some jitter (mean: 76.85 ms, SD: 6.57 ms) ranging from 60--100 ms. To optimize the setup for natural communication, audio signals can be delayed to match the latency of the video signals (white bar).

L297:

Discussion

We established an MEG hyperscanning system with an audiovisual interface capable of permitting real-time, face-to-face communication between two adults, and verified its TTL signaling and audiovisual transmission latency. The latency of the TTL signal (trigger) was orders of magnitude lower than the maximum temporal resolution of our MEG devices, essentially demonstrating simultaneous and synchronous recording onset for both MEG devices. Site-to-site audio signal latency was about 3 ms, which is on par with the speed of transmission of telephone landline audio signals[34]. Moreover, audio latency was completely jitter free, and well below reported thresholds for human detection of musical quality deterioration, indicating that our system would additionally be suitable for communication paradigms based on musical stimuli[44]. Finally, the video signals had short latencies (60--100 ms) and small jitter (SD:6.57 ms).

**- An electrophysiological experiment should be performed to use the hyperscanning system in practice. For example, audio, video, and audio-video stimuli are presented at the site A and transmitted to the site B. At the same time, MEG data of a participant are recorded at the site B and the data are analyzed. In this experimental paradigm, electrophysiological signals such as event-related potentials and event-related spectral perturbation for audio, video, and audio-video stimulus can be obtained and the results can verify the system.**

Thank you for this important suggestion. In response, we have appended the manuscript with data based on Event Related Desynchronization (ERD) recorded with our system during real-time, face-to-face conversation. For your convenience, the revised parts of the manuscript are shown below.

L190:

Electrophysiological experiment

One pair of subjects (23 year-old female and 25 year-old male) participated. Signed informed consent was obtained from both subjects before the experiment. The MEG recordings were approved by the Ethics Review Board of the Graduate School of Medicine at Hokkaido University. The two subjects faced each other via the A/V devices and spoke words in turns according to timed cues. The speech audio signals from each site were transmitted to the opposite site with a 90 ms delay using the ADL to align them with the visual signal delay. MEGs were recorded during 128 speech exchanges of this alternate speaking protocol. The amplitude modulations of the alpha-band rhythms across all 128 exchanges were averaged and then normalized in each subject based on their average alpha amplitude over the period from -2000 ms to -1000 ms prior to the speech onset of the other subject (S2 File). The resulting normalized alpha activity was then mapped onto template brains. Data analysis was performed with Brainstorm [45].

L275:

Electrophysiological experiment

Fig 3 shows normalized alpha-band amplitude modulation for each subject averaged across the entire cortical surface (Upper), and the across both subjects and mapped onto the template brain (Lower). The brain activity of the subjects at both sites reflects that which is associated with listening, with time point 0 ms being the moment of speech onset of the opposite party. Alpha-band desynchronization was exhibited in both the site A and site B subject during listening. Notably, the desynchronization appears to have commenced before the speech onset of the opposite party, a sign that subjects could visually predict the onset of the opposite party’s speech. The suppression was primarily concentrated in occipital and left temporal regions, indicating functional involvement of both the visual and auditory systems, and suggesting that each subject could visually predict the onset of the opposite party’s speech.

L287:

**Fig 3. Amplitude modulation of alpha-band rhythm during face-to-face conversation.** The two subjects faced each other via A/V devices and spoke words in turns. Brain activity shown is that which is associated with listening, with time point 0 ms being the moment of speech onset of the opposite party. Event Related Desynchronization (ERD) of alpha band is exhibited just before and during the speech of the opposite party (Upper: amplitudes were averaged over whole head of each subject), with involved regions observed mainly in occipital and left temporal region (Lower: mean ERS/D of the two subjects), indicating functional involvement of both the visual and auditory systems, and suggesting that each subject could visually predict the onset of the opposite party’s speech.

L309(discussion)

　We also conducted an experiment to detect electrophysiological responses and confirmed that this hyperscanning system can reliably transmit A/V information and measure physiological signals.
